# PROTOCOL: Risk and strength factors that predict criminal conduct among under‐represented genders and sexual minorities: A systematic review and meta‐analysis

**DOI:** 10.1002/cl2.1312

**Published:** 2023-03-02

**Authors:** Aminah Chambers, Shelley Brown, Michele Peterson‐Badali

**Affiliations:** ^1^ Applied Psychology & Human Development, Ontario Institute for Studies in Education University of Toronto Toronto Ontario Canada; ^2^ Department of Psychology Carleton University Ottawa Ontario Canada

## Abstract

This is the protocol for a Campbell systematic review. The objective of this review is to synthesize the evidence to identify risk and strength factors that predict the criminal offending in underrepresented genders and sexual minorities.

## 1 BACKGROUND

### 1.1 The problem, condition or issue

In the criminal justice literature, a well‐documented gender gap exists between crimes committed by women/girls and men/boys. Females offend at a lower rate than males^1^ and this gap is wider for violent crimes (Scott et al., 2019; Wattanaporn & Holtfreter, 2014). As a result, historically women and girls have not been the main focus of criminal justice research in studies about criminal conduct (Scott et al., 2019). Due to the gender gap, crimes committed by females were understudied and not well understood until feminist scholars began conducting female‐centered research in the ‘90s, resulting in gender‐responsive theories seeking to explain why girls and women engage in criminal conduct (Brown et al., 2019; Scott et al., 2019). An example of a gender‐responsive theory is pathways theory (Daly, 1992). Pathway theory proposes that some of the reasons men and women commit and desist from crime are different (Kruttschnitt, 2016; Scott et al., 2019). For example, trauma has been identified as playing a central role in the onset of offending for many women and girls (Pasko, 2008).

Many studies since the ‘90s have provided support for the theory of gender differences in risk factors for female offending. For example, a systematic review and meta‐analysis on male and female youth found that certain individual risk factors were *female‐specific*—predictive for girls and not for boys—such as truancy from school and out‐of‐home placement. Other variables were *female‐salient*—effects more pronounced for girls but still significant in boys—such as number of convictions, family substance abuse, and chronic alcohol use (Scott & Brown, 2018). However, some scholars (e.g., Burton et al., 1998) argue that both male and female crime can be described by gender‐neutral theories. Two gender‐neutral theories include: self‐control theory (irrespective of gender, crime is more likely to occur when people favor short‐term pleasure over long‐term consequences) and social learning theory (irrespective of gender, crime is learned through interactions with others (Gottfredson & Hirschi, 2010; Pratt et al., 2010). As well, there is empirical evidence that some risk factors are gender neutral—predictive for both males and females, to the same degree (Gottfredson & Hirschi, 2010; Olver et al., 2014; Pratt et al., 2010; Scott & Brown, 2018).

Traditionally risk assessment has operated from a deficit‐based framework which entails identification of risk factors and treatment of criminogenic needs in correctional settings (Jones et al., 2016). However, the concept of strengths, based in positive psychology, resilience, and criminal desistance (Brown et al., 2020), may be equally important in understanding onset (i.e., what factors predict initial involvement in criminal/antisocial conduct) and/or desistance (ceasing involvement in criminal activity), particularly in females. For the purposes of this paper, strengths are conceptualized as positive attributes of an individual and/or their environment regardless of their empirical association with judicial outcomes. Judicial outcomes include a range of outcomes such as initial sentencing in a court of law, or criminal re‐arrests or reconvictions post release after an original sentence (Brown et al., 2020; Jones et al., 2015). Promotive factors are strengths that negatively predict justice outcomes irrespective of risk level (Brown et al., 2020; Farrington et al., 2016). Protective factors are strengths that negatively predict justice outcomes with stronger effects observed in high‐risk people (Brown et al., 2020; Farrington et al., 2016).

The area of underrepresented genders and sexual minorities is another understudied area in the field of criminal offending, but research is emerging. A 2019 meta‐analysis found that sexual minority girls were more than twice as likely to be involved in the justice system than their heterosexual peers (Jonnson et al., 2019). Other studies have shown sexual minority girls are more likely to be stopped by police (Himmelstein & Brückner, 2011).

Researchers have hypothesized several reasons for the higher rates of justice involvement in sexual minorities in general (e.g., 13% vs. 5% in the youth general population; Conover‐Williams & Teal, 2019). First, the criminalization of survival behaviors, such as shoplifting or sex work have been identified as plausible explanations for both sexual minorities and cisgender girls and women (Chesney‐Lind & Pasko, 2013; Conover‐Williams & Teal, 2019). Second, sexual minorities may also have different factors that contribute to offending, such as greater rates of homelessness (due to housing instability), victimization, or marginalization (Conover‐Williams & Teal, 2019). Research on LGBTQ + youth in juvenile courts has indicated that leaving home (e.g., running away or being kicked out) due to family rejection is “the greatest predictor of future involvement with the juvenile justice system for LGBT youth” (Majd et al., 2009, p. 72).

A third paradigm that may explain gender/sexual minority differences in crime is developmental life course criminology, which suggests that multiple factors, including gender, may influence the “pushes and pulls toward/from offending” (Conover‐Williams, 2014, p. 451). The onset to offending for underrepresented genders and sexual minorities may also differ from what's found in the male‐dominated literature. Pathways theory proposes five paths to offending for cisgender women and girls, including life experiences such as having been abused or neglected as a child, running away from home and engaging in crime to survive, domestic violence survivors, or drug addiction (Daly, 1992; Gehring, 2018). These categories overlap with risk factors identified for sexual minority women and girls, such as victimization or histories of abuse (Belknap et al., 2012). Identifying risks and strengths that may be gender‐neutral versus gender‐salient/specific or potentially LGBTQIA + salient or specific is critical to the development of interventions and risk assessment tools that are both inclusive and evidence based.

Before the rise of gender responsive literature, most assessment and intervention historically focused on males with little consideration for gender differences in reasons for offending or offending patterns (van Voorhis et al., 2008). The Level of Service Inventory—Revised (and its variants) is a commonly used correctional instrument to assess an individual's risk to reoffend (LSI‐R; Andrews et al., 1995). Reisig et al. (2006) found the LSI‐R did not predict risk and needs as well for some women as it did for men, and that it performed particularly poorly when women had gendered pathways of crime identified under pathways theory, such as drug‐connected women. In contrast, others have found that the LSI family of instruments predicts recidivism equally well for men and women (Olver et al., 2014; Vose et al., 2009).

Feminist scholars have expressed concerns that simply controlling for gender in studies may miss female‐specific patterns in offending. They propose rather than simply controlling for gender, researchers should (1) design studies and risk assessment tools with a gender‐responsive lens from inception and (2) disaggregate analyses by gender to further understand the unique contributors to crime for women (Wattanaporn & Holtfreter, 2014). Feminist scholars have created gender‐responsive risk assessment supplements to address these issues for cisgender women (e.g., Van Voorhis et al., 2010), but no tools to date address gender/sexual diversity.

A better understanding of the relative importance of factors for all genders/sexual identities begins with identifying potentially understudied (and commonly studied) variables in samples of underrepresented genders and sexual minorities. Thus, this research will seek to identify potential risk and strength factors in underrepresented genders (i.e., girls/women/trans/nonbinary) and sexual minorities to explore which factors are most predictive of criminal offending in these groups. Further, the variables of interest will be drawn from both the gender responsive and the gender‐neutral literature bases. The gender responsive literature has examined hypothesized female‐specific variables (e.g., abuse, mental health, self‐esteem, relational aggression) in samples of predominately females. In contrast, the gender‐neutral literature has focused on hypothesized gender‐neutral variables (e.g., criminal history, criminal attitudes, criminal associates, substance use, unemployment) in samples of predominately males. Hypothesized female‐specific variables have garnered little attention from gender‐neutralscholars. Similarly, hypothesized gender neutral factors have garnered little attention from gender‐responsive scholars.

### 1.2 Outcome

Outcomes will include official criminal offending, including non‐violent offending, violent offending (Olver et al., 2011), sexual offending, or nonsexual offending (Hanson and Bussiere, 1998). We will also include any type of unofficial offending, self‐reported delinquency, antisocial behavior, or crime that could result in an arrest if caught (e.g., disorderly conduct, stealing, carrying a weapon; Piquero et al., 2002). For youth, status offenses, defined as conduct that would not be considered a crime for adults (e.g., skipping school, running away; Office of Juvenile Justice and Delinquency Prevention, 2014) will be included. Each type of offending (i.e., official, unofficial, and status offenses) will be reported and analyzed separately where possible.

### 1.3 Risk/strength factors

Possible risk/strength factors salient to cisgender women and girls such as leisure time, self‐esteem, or childhood maltreatment have been identified in prior research (e.g., Brown et al., 2019; Scott & Brown, 2018). Conover‐Williams (2014) found certain risk factors (such as victimization and housing instability) and strength factors (such as school and family attachments) may be more predictive for sexual minorities; this research was not disaggregated for cisgender women. Based on past meta‐analyses (e.g., Hubbard & Pratt, 2002; Scott & Brown, 2018) and consistent with theories such as pathways theory, we anticipate using the following category risk labels: criminal history, family criminality, employment/education, substance use, antisocial peers, use of leisure time, antisocial personality, antisocial attitudes, abuse, trauma/PTSD, victimization and the following strengths: family support, religiosity, cognitive abilities (e.g., intelligence, executive functioning), self‐concept, and peer support, but there may more and/or these may change as a function of the search. As this is a systematic review on risk and strength factors, we will include and report on any factors found. No factors located in the literature will be excluded.

### 1.4 How the risk/strength factors might be linked to the outcome

*Risk factors* are defined as “a characteristic, experience, or event that, if present, is associated with an increase in the probability (risk) of a particular outcome over the base rate of the outcome in the general (unexposed) population” (Haggerty & Mrazek, 1994; Kazdin et al., 1997). In this study, a risk factor specifically refers to the likelihood of criminal justice involvement/offending. Strengths are any conceptual positive attributes of an individual and/or their environment regardless of empirical judicial outcomes, that is, “a positive or prosocial facet of an individual's life… (p. 323; Brown et al., 2020; Jones et al., 2015).” We intend to further categorize strength factors as promotive (negatively correlated with justice outcomes irrespective of risk level) and, depending on whether studies report risk levels (e.g., high risk, low risk), or protective (negatively correlated with justice outcomes with stronger effects in high‐risk individuals (Brown et al., 2020; Farrington et al., 2016).

### 1.5 Why it is important to do the review

Criminological scholarship has historically focused primarily on cisgender male samples. As a result, risk and strength factors for males are well documented. However, these factors are less clear for underrepresented genders and sexual minorities. Consequently, most assessment and intervention approaches are grounded in male‐focused research with little consideration for potential gender and/or sexuality differences. Many scholars have argued that this practice has led to the over‐classification of cisgender girls/women in correctional settings and a failure to identify and hence address the needs that are hypothesized to be more salient among justice‐involved female populations (van Voorhis et al., 2008). The majority of scholars agree on factors that contribute to offending for all people, but the relative importance of risk (and strength) factors as well as prioritization in treatment may be different for underrepresented genders and/or sexual minorities.

In 2010, the United Nations unanimously agreed on 70 gender‐based rules for women in correctional settings, known as the Bangkok Rules (United Nations, 2011). The guiding principle of the rules is that while women are a small minority of the world's incarcerated population, they have unique needs within correctional settings and different factors that lead to their involvement in the justice system. In the age of evidence‐based practice, more evidence is needed that focuses on the needs, strengths, and risk factors for underrepresented genders/sexual minorities to ensure equitable treatment in the criminal justice system. Consistent with the Bangkok rules and other human rights initiatives (e.g., Gender‐Based Analysis Plus, Status of Women Canada, 2017) policymakers and organizations serving the justice sector are increasingly interested in how gender/sexual minority differences affect criminal offending, as well as how gender differences affect responses to interventions (e.g., Brown et al., 2019). A better understanding of risks and strengths applicable to underrepresented genders and sexual minorities provides a foundation for criminal justice policies, interventions, and assessments that meet the needs of these populations. This systematic review/meta‐analysis will report on the relative importance of the risk and strength factors for the onset of criminal offending in underrepresented genders and sexual minorities.

## 2 OBJECTIVES

The objective of this review is to synthesize the evidence to identify risk and strength factors that predict the criminal offending in underrepresented genders and sexual minorities. Research questions include:

1. What risk factors predict the initial onset of offending in cisgender women and girls, and are the risk factors different for cisgender girls and women?
2. What risk factors predict the onset of offending in trans/nonbinary people and sexual minorities? Are there differences between adult and youthful trans/nonbinary people and/or sexual minorities?
3. Are there differences in the risk factors that predict the onset of offending for cisgender women/girls in comparison to trans/nonbinary people and/or sexual minorities?
4. What strength factors negatively predict the onset of offending in cisgender women and girls, and are the strength factors different for girls and women?
5. What strength factors negatively predict the onset of offending in trans/nonbinary people and/or sexual minorities, and do the strength factors differ between adult and youthful trans/nonbinary people and/or sexual minorities?

## 3 METHODS

### 3.1 Criteria for considering studies for this review

#### 3.1.1 Types of studies

Study design will be limited to quantitative longitudinal studies that include a measure of risk and/or strength and a measure offending: official or unofficial criminal behavior, or status offending (in youth). Longitudinal studies can be either prospective or retrospective. Qualitative‐only studies will be excluded. Theoretical or conceptual articles (e.g., de Vogel & Nicholls, 2016) will be excluded.

#### 3.1.2 Types of participants

The population of interest will include cisgender women/girls, transgender women/girls, transgender men/boys, non‐binary/gender non‐conforming people (Crossley, 2019), and sexual minorities (lesbian, gay, bisexual, asexual, pansexual, etc.) No restrictions will be placed on geographic location or other demographic factors. Studies with male‐only populations will be excluded if they do not include a sexual minority sample. This study does not seek to directly test for differences between male and females. The goal is to identify risk and strength factors that predict future delinquency and/or criminal offending in samples of underrepresented genders and sexual minorities. Studies that include both males and females will be included if results are disaggregated by gender such that the outcomes of females only can be determined.

#### 3.1.3 Risk/strength predictive factors

No predetermination will be made as to eligible factors; however, all potential factors must be measured before the outcome (Kraemer et al., 1997). Based on a previous review of gender‐based risk/strength factors (Brown et al., 2019), factors that may be located in the literature include (this list is not exhaustive):

Individual characteristics: executive functioning, stress, self‐concept, education, use of leisure time, age, race/ethnicity, criminal history, age at first offense, offense type, intelligence. Examples may include scores on risk/needs assessments, IQ test scales, and results from educational assessments.

Social factors: childhood maltreatment, out‐of‐home placement, runaway, parental supervision, parent‐child relationship, family criminality, living with partner, relationship quality, recent abuse, antisocial peers, peer support, community level factors, discrimination, religiosity. Examples may include reports from child welfare agencies, results from self‐report parenting questionnaires, or results from self‐reported discrimination questionnaires.

Economic factors: employment, community level factors, poverty, deprivation, socioeconomic class. Examples may include socioeconomic factor indexes or self‐reported employment earnings.

Psychological factors: criminal thinking, anti‐social personality, externalizing, internalizing, major mental illness, substance use (Brown et al., 2019; Wolfowicz et al., 2020). Examples may include results from risk/needs assessments, clinician‐administered mental health assessments, or substance abuse screening results.

#### 3.1.4 Types of outcome measures

The outcome of interest will be any measure of offending: official or unofficial criminal conduct, or status offending (in the case of youth). Any time range of reoffending (e.g., 1 year, 3 years, etc.) will be included. All types of reporting will be included: self‐report, third‐party, and official reports of justice‐system involvement (Scott & Brown, 2018).

#### 3.1.5 Types of settings

The study will include underrepresented genders and sexual minorities in three areas: general population, at‐risk samples, or justice‐involved (incarcerated, probation, parole, mental health courts, drug courts, or other diversion programs). No restrictions will be placed on locations of studies (i.e., country of origin).

### 3.2 Search methods for identification of studies

#### 3.2.1 Electronic searches

We will use Zotero to manage references. Sources searched, name of researcher performing the search, and date of search will be documented. Searches older than 12 months from the intended publication date will be re‐run to identity and include new potentially eligible studies.

Search terms

1. Offending
  recid* or arrest* or offend* or reoffen* or convict* or reconvict* or adjudic* or delinq* or re‐entry or crime or criminal* or parole* or probation* or feminine* or criminogenic* or incarcerat* or detain or court* or divert or diversion* or “justice system” or antisocial
  AND
2. Risk/Strength
  a. (risk* adj3 recid*) or (risk* adj3 offend*) or (risk* adj3 reoffen*) or (risk* adj3 criminogenic) or ((risk* adj3 delinq*) or (factor* adj5 recid*) or (factor* adj5 offend*) or (factor* adj5 reoffen*) or (factor* adj5 criminogenic) or (factor* adj5 delinq*) or “risk factor*” or “risk scale*” or “at‐risk” or “cox regression*” or “cox proportional hazard*” or “proportional hazard*” or “survival analys*” or “logistic regression” or longitudinal or cohort* or “risk model*”
    OR
  b. strength* OR protective* OR promotive OR buffer* OR resilien*
  AND
3. Gender
  a. gender* OR girl* OR female* OR women* OR woman OR feminine*
    OR
  b. “trans” OR transgender OR transsexual OR bisex* OR androg* OR gay* OR queer* OR lesbian* OR homosexual* OR asexual* OR genderfluid* OR intersex* OR pansexual* OR third‐gender OR two‐spirit OR “sexual minorit*” OR questioning OR “gender non‐conforming” OR LGB*

The search strategy will be applied to the following databases, gray literature sources, and relevant journals not included in the databases; it will also be customized to each platform (Brown et al., 2019; Higginson et al., 2018). All journals listed will be hand searched by accessing the search function when available and reading through the index of all identified journals when a search function is not available.

*Databases*

Criminal Justice Abstracts with Full Text (EBSCO)
Child and Adolescent Studies (EBSCO)
Gender Studies (EBSCO)
Education Source (EBSCO)
ERIC (ProQuest)
Sociological Abstracts (ProQuest)
Applied Social Sciences Index (ProQuest)
Criminal Justice Database (ProQuest)
PsycINFO (OVID)
MEDLINE (OVID)
EMBASE (OVID)
CINAHL (EBSCO)
PsycEXTRA
International Bibliography of the Social Sciences (ProQuest)
Social Sciences Citation Index (Web of Science)
Conference Proceedings Index (Social Science; Web of Science)
Emerging Sources Index (Web of Science)

*Gray Literature*

Dissertations and Theses (ProQuest)
Correctional Service of Canada
Public Safety Canada
Elizabeth Fry Society
Australian Institute of Criminology
National Criminal Justice Reference Service
U.S. National Institute of Corrections
Bureau of Prisons
U.K. Home Office
U.K. Ministry of Justice
The Urban Institute
The Pew Charitable Trust
The Center for Gender and Justice
Women's Prison Association
RAND

*Journals*

*Pakistan Journal of Criminology*
*African Journal of Criminology and Legal Studies*
*Asian Journal of Criminology*
*Indian Journal of Criminology*
*South African Journal of Criminal Justice*
*South African Crime Quarterly*
*Turkish Journal of Criminology*
*LILACS*
*International Juvenile Justice Observatory*
*Don M. Gottfredson Library of Criminal Justice*
*Campbell Systematic Reviews*
*Criminology*
*Feminist Criminology*
*Journal of Developmental and Life‐Course Criminology*
*Journal of Quantitative Criminology*
*Women and Criminal Justice*

#### 3.2.2 Searching other resources

We will perform reference harvesting on prior reviews such as Brown et al. (2019) and Scott and Brown (2018). We will search the reference lists of all included studies and contact experts in the area known to us for any in press, recently published, or unpublished relevant works (Windisch et al., 2020). No restrictions will be placed on publication status or language of publication. We will include dates of searches, databases searched (including platform), number of results returned, reasons for exclusion of studies that were included in the full‐text review, and a PRISMA flowchart. We will document authors contacted and results, including dates and number of attempts (Windisch et al., 2020).

### 3.3 Data collection and analysis

#### 3.3.1 Selection of studies

We will single screen studies with DistillerSR using the following criteria:

a. Does the study mention a justice‐related outcome?
b. Does the study mention females, a comparison of females and males, or a gender or sexual minority population?
c. Does it seem plausible that the study reports risk and/or strength factors?

Studies that meet all criteria will be included in the full‐text screening (Windisch et al., 2020). If changes are made to eligibility criteria, study authors will agree upon new eligibility criteria and document in the review any changes made from the protocol (Windisch et al., 2020).

#### 3.3.2 Data extraction and management

A data extraction form will be created and managed in DistillerSR (see Online Supplements). After piloting the form to ensure consistency, two independent authors will extract data from the studies, with discrepancies resolved either by agreement or with a third party (Windisch et al., 2020). We will describe the study according to the Methodological Expectations of Campbell Collaboration Intervention Reviews (MECCIR) reporting standards, including information on type of publication, country of origin, sample descriptives (e.g., number of males, females) types of crimes, risk level, outcome characteristics, predictors, effect sizes, etc. (Scott, 2017; Windisch et al., 2020), and we will code multiple effect sizes if present (Wilson et al., 2018).

To avoid double counting, if multiple publications by the same authors of the same data set are found with the same risk/strength independent variable(s) and outcome combination(s), we will code the study with the most information as the primary study. Any others will only be used for cross‐referencing (Wilson et al., 2018). If the primary study includes multiple outcomes with the same risk/strength independent variable(s) combination(s), we will code all effect sizes found but follow these decision rules:

(1) We will prioritize raw coefficients over covariate‐adjusted estimates, but we will run secondary analyses for covariate‐adjusted estimates.
(2) We will prioritize the highest‐ranking outcomes as follows: (1) official, (2) unofficial, and (3) status offenses. We will perform supplementary analyses (e.g., replicate analyses for unofficial and status offenses if more than two effect sizes are found) for each type of outcome.
(3) We will prioritize the most serious outcome (e.g., within the official outcome category if arrests and convictions are reported, we will prioritize convictions; Scott, 2017) and perform supplementary analyses if more than two effect sizes are found.
(4) Lastly, if studies report multiple effects for different follow‐up periods, the study with the longest length of time after index offense will be selected (Feder & Wilson, 2006).

#### 3.3.3 Assessment of risk of bias in included studies

Studies will be assessed for bias using a risk assessment tool included in the supplemental material (Higginson et al., 2014). All full‐text eligible studies will be reviewed independently by at least two authors. Disagreement will be resolved by consulting a third reviewer.

#### 3.3.4 Measures of predictors/risk factors

We anticipate predictors will be captured by: (1) standardized self‐report questionnaires, (2) non‐standardized self‐report, (3) risk assessments and (4) file review/third party report.

#### 3.3.5 Measures of the outcome

Outcomes are expected to be both dichotomous and continuous and are anticipated to come from self‐report questionnaires, official/government outcomes, self‐report/unofficial offending, and family reports. Different types of effect sizes will be statistically converted to correlations and pooled where possible (Wolfowicz et al., 2020)^2^. We will ensure the direction of scores on combined scales have the same meaning/directionality and will report when scales are reversed. Before meta‐analysis, we will check for skewness in outcome variables and transform as necessary (Windisch et al., 2020).

#### 3.3.6 Unit of analysis issues

The unit of analysis is an independent effect size. If we still have non‐independent effect sizes at this stage (e.g., two measures of family support predicting the same outcome in the same sample) we will first code the subordinate categories. If there are at least five effect sizes, we will use Tipton's (2015) small sample adjustment approach to adjust for effect size dependence. If there happens to be more than 40 effect sizes (unlikely) we will use the original robust variance estimation method (Hedges et al., 2010). If there are less than five effect sizes, we will take the weighted average.

#### 3.3.7 Dealing with missing data

When data are missing, we will first make reasonable inferences about the missing data where possible (Pigott & Polanin, 2020). Second, we will attempt to collect missing data from authors. If this is not possible and if the data are missing‐at‐random, we will use multiple imputation for missing moderator data, but we will not impute effect sizes (Pigott & Polanin, 2020). The amount and type of missing data will be reported in the risk of bias analyses (Windisch et al., 2020).

#### 3.3.8 Assessment of heterogeneity

We will assess for heterogeneity using *I* ^2^ in combination with *τ* ^*2*^ and *χ* ^*2*^ (Windisch et al., 2020).

#### 3.3.9 Assessment of reporting biases

To determine if selective reporting bias is present, we will create funnel plots and check for funnel plot asymmetry using Egger's test (Egger et al., 1997), and Duval & Tweedie's trim‐and‐fill procedure (Duval & Tweedie, 2000).

#### 3.3.10 Data synthesis

We expect to find effect sizes of various combinations of continuously and dichotomously scored IVs and DVs. But continuously, or dichotomously scored IVs paired with dichotomously scored outcomes are expected to be the most common. As a result, we will use the correlation coefficient as our effect size of choice when one or both variables are measured continuously. In the case of dichotomously scored IV and DV pairs, we will calculate a correlation coefficient if the requisite information is provided (e.g., raw cell counts, or *χ* ^2^ values and sample sizes (Wilson, n.d.). If we are required to convert an odds ratio to a correlation coefficient (due to the absence of requisite information) we will adopt Borenstein et al.'s (2009) approach (e.g., log odds ratio to Cohen's *d* to *r*). In sum, different types of effect sizes will be statistically converted to correlations and pooled where possible (Wolfowicz et al., 2020). We will analyze raw covariates and covariate‐adjusted estimates separately. Meta‐analysis will be conducted using a restricted maximum likelihood random effects model in R (Harrer et al., 2021).

### 3.4 Subgroup analysis and investigation of heterogeneity

Subgroup analyses will be performed on age (e.g., youth, adult), cisgender women/girls versus other underrepresented genders/sexual minorities, custody status [i.e., at‐risk, community‐based justice involved (e.g., probation), or incarcerated], race/ethnicity, severity of index crime, and risk level. To determine differential outcomes using direct tests of mean differences between the groups, we will use the random effects analog‐to‐the‐ANOVA method for single categorical variables and random effects meta‐analytic regression methods for continuous moderators (Wilson et al., 2018). We will assess for heterogeneity using the *I* ^2^ in combination with *τ* ^*2*^ and *χ* ^*2*^ (Windisch et al., 2020).

#### 3.4.1 Sensitivity analysis

Where multiple imputation is used to account for missing data, a complete case analysis will be completed first and then compared to the imputed data. (Jakobsen et al., 2017; Pigott & Polanin, 2020).

## ROLES AND RESPONSIBILITIES

Content: Dr. Shelley Brown has been conducting quantitative research in the realm of corrections for over 20 years and qualitative research for five years. Between 1997 and 2006, Brown worked for the Correctional Service of Canada conducting applied research in the area of risk assessment and program evaluation including revisions to the Dynamic Factor Identification and Analysis tool (DFIA‐R) (Brown & Motiuk, 2005). The revisions involved streamlining the DFIA‐R and making it more responsive to women and Indigenous peoples. Dr. Brown currently works as an Associate Professor in the Department of Psychology with a research focus on improving gender responsive services for girls and women in the criminal justice system using a mix of quantitative and qualitative approaches. She also actively incorporates male comparison groups in her research to enable stronger conclusions regarding gender specificity. More recently, Dr. Brown has become interested in researching complex trauma, violence, strengths, risk assessment and desistance among justice‐involved girls and women with her various collaborators from the Centre for Addiction and Mental Health (CAMH) and Orbis Partners. Dr. Brown's work is primarily funded by the Social Sciences and Humanities Research Council (SSHRC).

Dr. Michele Peterson‐Badali's research focuses on children's and adolescents' knowledge, reasoning, perceptions, and experiences of the youth justice system, their understanding of rights, and their evolving legal capacities. One of the guiding principles of her research program over the past 30 years has been to provide an evidence basis for Canadian youth justice policy and practice. Past research has examined adolescents' understanding of due process rights; youths' knowledge of Canadian youth justice legislation; perceptions and experiences of peer violence in youth custody settings; and parental involvement in the youth justice system. Current research addresses effective rehabilitation for justice‐involved youth in the context of the risk‐need‐responsivity framework, including a focus on Indigenous youth; and the role of mental health issues in youth justice system involvement.

Aminah Chambers is a 4th‐year PhD student in the Youth Justice Lab under the supervision of Dr. Michele Peterson‐Badali at the University of Toronto/OISE. Using both qualitative and quantitative methods, she is currently examining court processes for youth and their families who encounter the justice system, and ways that courts can identify and address the complex needs of youth with mental health issues.

Systematic review methods: Dr. Brown has conducted and published meta‐analyses and systematic reviews (e.g., Brown et al., 2019; Scott & Brown, 2018). Aminah Chambers has completed coursework on systematic review and meta‐analysis, assisted with systematic review (Brown et al., 2019) and has consulted extensively with research librarians at the University of Toronto's Gerstein Science Information Centre.

Statistical analysis: Aminah Chambers has completed coursework in advanced multivariate statistical analysis, systematic review, and meta‐analysis.

Information retrieval: Aminah Chambers completed coursework in systematic review and meta‐analysis and also consulted extensively with research librarians at the University of Toronto's Gerstein Science Information Centre for this project.

## SUPPORT OF SOURCES

No financial support or funding to declare. No funding applications are planned with regard to this project.

## DECLARATIONS OF INTEREST

Shelley Brown is a co‐author of potentially five or more studies that fall within the scope of this review and meta‐analysis. Michele Peterson‐Badali is a co‐author of one study that falls within the scope of this review and meta‐analysis and was therefore potentially eligible for inclusion in the review.

## PLANS FOR UPDATING THE REVIEW

The review will be updated every 5 years by Aminah Chambers and/or Shelley Brown.

## Supporting information

Supporting information.

Supporting information.

## Footnotes

1 In this paper, the term “female” refers to both girls and women; “males” refers to both boys and men. “Underrepresented genders” refers to cisgender women/girls, transgender women/girls, transgender men/boys, and non‐binary/gender non‐conforming youth or adults. “Sexual minority” refers to all sexual identities other than heterosexual.

2 For further explanation of techniques described in this paper, the following books are provided for reference: (1) Borenstein, M., Hedges, L., Higgin, J., & Rothstein, H. (2011). *Introduction to meta‐analysis*. John Wiley & Sons, Inc. and (2) Lipsey, M. W., & Wilson, D. B. (2009). *Practical meta‐analysis*. Sage Publications.
