# Supplementary material for: PROTOCOL: Risk and strength factors that predict criminal conduct among under‐represented genders and sexual minorities: A systematic review and meta‐analysis
Source: Campbell Syst Rev. 2023 Mar 2;19(1):e1312. doi: 10.1002/cl2.1312 (PMC9979961; doi:10.1002/cl2.1312)
Supplement: Supplementary file 1 — Supporting information. [file CL2-19-e1312-s001.docx]

Appendix A

Search terms

1. Offending

recid* or arrest* or offend* or reoffen* or convict* or reconvict* or adjudic* or delinq* or re-entry or crime or criminal* or parole* or probation* or sentenc* or criminogenic* or incarcerat* or detain or court* or divert or diversion* or "justice system" or antisocial

AND

1. Risk/Strength
   1. (risk* adj3 recid*) or (risk* adj3 offend*) or (risk* adj3 reoffen*) or (risk* adj3 criminogenic) or ((risk* adj3 delinq*) or (factor* adj5 recid*) or (factor* adj5 offend*) or (factor* adj5 reoffen*) or (factor* adj5 criminogenic) or (factor* adj5 delinq*) or "risk factor*" or "risk scale*" or "at-risk" or "cox regression*" or "cox proportional hazard*" or "proportional hazard*" or "survival analys*" or "logistic regression" or longitudinal or cohort* or "risk model*")

OR

- 1. strength* OR protective* OR promotive OR buffer* OR resilien*

AND

1. Gender
   1. gender* OR girl* OR female* OR women* OR woman OR feminin*

OR

- 1. "trans" OR transgender OR transsexual OR bisex* OR androg* OR gay* OR queer* OR lesbian* OR homosexual* OR asexual* OR genderfluid* OR intersex* OR pansexual* OR third-gender OR two-spirit OR "sexual minorit*" OR questioning OR "gender non-conforming" OR LGB*

**Database:**
APA PsycInfo <2002 to July Week 2 2022>

| **#** | **Query** | **Results from 15 Jul 2022** |
| --- | --- | --- |
| 1 | exp juvenile delinquency/ | 10,568 |
| 2 | exp Criminal Offenders/ | 14,000 |
| 3 | exp Crime/ | 89,478 |
| 4 | exp Criminal Justice/ | 12,232 |
| 5 | exp Recidivism/ | 4,995 |
| 6 | exp Defendants/ | 1,133 |
| 7 | exp Adjudication/ | 11,295 |
| 8 | exp Juvenile Justice/ | 3,099 |
| 9 | exp Correctional Psychology/ | 85 |
| 10 | (recid* or arrest* or offend* or reoffen* or convict* or reconvict* or adjudic* or delinq* or re-entry or crime or criminal* or parole* or probation* or sentenc* or criminogenic* or incarcerat* or detain or court* or divert or diversion* or "justice system" or antisocial).tw. | 142,278 |
| 11 | 1 or 2 or 3 or 4 or 5 or 6 or 7 or 8 or 9 or 10 | 195,630 |
| 12 | exp Protective Factors/ | 6,601 |
| 13 | exp At Risk Populations/ | 25,836 |
| 14 | exp Risk Assessment/ | 13,008 |
| 15 | exp Risk Factors/ | 90,543 |
| 16 | exp "Resilience (Psychological)"/ | 17,606 |
| 17 | ((risk* adj3 recid*) or (risk* adj3 offend*) or (risk* adj3 reoffen*) or (risk* adj3 criminogenic) or ((risk* adj3 delinq*) or (factor* adj5 recid*) or (factor* adj5 offend*) or (factor* adj5 reoffen*) or (factor* adj5 criminogenic) or (factor* adj5 delinq*) or "risk factor*" or "risk scale*" or "at-risk" or "cox regression*" or "cox proportional hazard*" or "proportional hazard*" or "survival analys*" or "logistic regression" or longitudinal or cohort* or "risk model*")).tw. | 355,650 |
| 18 | (strength* or protective* or promotive or buffer* or resilien*).tw. | 194,600 |
| 19 | 12 or 13 or 14 or 15 or 16 or 17 or 18 | 546,013 |
| 20 | exp Female Criminal Offenders/ | 1,182 |
| 21 | exp Female Delinquency/ | 303 |
| 22 | exp human sex differences/ | 68,109 |
| 23 | exp Human Females/ | 96,312 |
| 24 | exp LGBTQ/ | 26,597 |
| 25 | exp Gender Identity/ | 32,654 |
| 26 | exp Lesbianism/ | 9,179 |
| 27 | exp Bisexuality/ | 7,511 |
| 28 | exp Transsexualism/ | 1,996 |
| 29 | exp Transgender/ | 7,028 |
| 30 | exp Sexual Orientation/ | 33,019 |
| 31 | (gender* or girl* or female* or women* or woman or feminin*).tw. | 537,026 |
| 32 | ("trans" or transgender or transsexual or bisex* or androg* or gay* or queer* or lesbian* or homosexual* or asexual* or genderfluid* or intersex* or pansexual* or third-gender or two-spirit or "sexual minorit*" or questioning or "gender non-conforming" or LGB).tw. | 55,318 |
| 33 | 20 or 21 or 22 or 23 or 24 or 25 or 26 or 27 or 28 or 29 or 30 or 31 or 32 | 590,246 |
| 34 | 11 and 19 and 33 | 14,583 |
| 35 | Incorporating Strengths Into Quantitative Assessments of Criminal Risk for Adult Offenders The Service Planning Instrument.m_titl. | 1 |
| 36 | (Risks, strengths, gender, and recidivism among justice-involved youth: A meta-analysis).m_titl. | 1 |
| 37 | women's offending behavior evidence based review of gender differences.m_titl. | 1 |
| 38 | are the major risk need factors.mp. [mp=title, abstract, heading word, table of contents, key concepts, original title, tests & measures, mesh word] | 1 |
| 39 | factors related to recidivism among delinquent females.m_titl. | 0 |
| 40 | gender differences in life-course theory of recidivism.m_titl. | 1 |
| 41 | using dynamic factors to predict recidivism among women.m_titl. | 1 |
| 42 | gender differences in recidivism rates for juvenile justice youth.m_titl. | 1 |
| 43 | girls will be girls gender differences in predictors of success.mp. [mp=title, abstract, heading word, table of contents, key concepts, original title, tests & measures, mesh word] | 1 |
| 44 | Classifying juvenile offenders according to risk of recidivism.m_titl. | 1 |
| 45 | 35 or 36 or 37 or 38 or 39 or 40 or 41 or 42 or 43 or 44 | 9 |
| 46 | 34 and 45 | 8 |
| 47 | (social functioning victimization and mental health).m_titl. | 1 |
| 48 | racial differences in the associations of neighborhood disadvantage.m_titl. | 1 |
| 49 | young adult outcomes of juvenile court involved girls.m_titl. | 2 |
| 50 | delinquent girls grown up young adult offending patterns.m_titl. | 1 |
| 51 | 35 or 36 or 38 or 39 or 40 or 41 or 42 or 43 or 44 or 47 or 48 or 49 or 50 | 13 |
| 52 | 34 and 51 | 13 |
|  |  |  |
