# Supplementary material for: PROTOCOL: Risk and strength factors that predict criminal conduct among under‐represented genders and sexual minorities: A systematic review and meta‐analysis
Source: Campbell Syst Rev. 2023 Mar 2;19(1):e1312. doi: 10.1002/cl2.1312 (PMC9979961; doi:10.1002/cl2.1312)
Supplement: Supplementary file 2 — Supporting information. [file CL2-19-e1312-s002.docx]

Online supplements

List of online supplements

1. Data extraction and codebook

**Extraction and coding sheet (Higginson et al., 2018; Scott & Brown, 2018; Wolfowicz et al., 2020)**

|  | |  |  |  |  |
| --- | --- | --- | --- | --- | --- |
|  | | **Category** | **Data type** | **Selection/Data entry** | **Comments/ Notes** |
| Publication information | Study ID  (cross-reference if necessary) | | Numeric |  |  |
|  | | 1. Author(s) name(s) | Text |  |  |
|  | | 2. Title | Text |  |  |
|  | | 3. Year | Number |  |  |
|  | | 4. Full reference | Text |  |  |
|  | |  |  |  |  |
|  | | Type: | Categorical |  |  |
|  | | a. Journal article (peer reviewed) |  |  |  |
|  | | b. Book |  |  |  |
|  | | c. Government report |  |  |  |
|  | | e. Police report |  |  |  |
|  | | f. Technical report |  |  |  |
|  | | g. Conference paper |  |  |  |
|  | | h. Dissertation or thesis |  |  |  |
|  | | J. Other (specify) |  |  |  |
|  | |  |  |  |  |
|  | | Publication details (complete for each study reported) |  |  |  |
|  | | 5. Location/country | Text |  |  |
|  | | 6. Language | Text |  |  |
|  | | 7. Date(s) of research | Number |  |  |
|  | | a. Start: | Date |  |  |
|  | | b. Finish: | Date |  |  |
|  | | 8. Source of funding | Categorical |  |  |
|  | | a. Government |  |  |  |
|  | | b. Foreign government |  |  |  |
|  | | c. Local university/research body |  |  |  |
|  | | d. Foreign university/research body |  |  |  |
|  | | e. NGO |  |  |  |
|  | | f. Other |  |  |  |
|  | | 9. Bodies involved (tick all applicable) | Categorical |  |  |
|  | | a. University/research agency |  |  |  |
|  | | b. Health Service |  |  |  |
|  | | c. Police/ Justice system |  |  |  |
|  | | d. Other government departments |  |  |  |
|  | | e. Other |  |  |  |
|  | | 10. Evaluated by | Text |  |  |
|  | | 11. Purpose of study to predict recidivism/offending? | Y/N |  |  |
|  | | 12. Ethical issues (Y/N. If yes, describe)? | Y/N |  |  |
|  | |  |  |  |  |
| Methodology | |  |  |  |  |
|  | | 13. Total number of cis females | Numeric |  |  |
|  | | 14. Total number of cis males  15. Total number of gender/sexual minorities  (provide details in comments) | Numeric  Numeric  Numeric  Numeric |  |  |
|  | | 16. Community based or incarcerated | Categorical |  |  |
|  | | a. Community |  |  |  |
|  | | b. Custody |  |  |  |
|  | | c. Mixed |  |  |  |
|  | | d. N/R |  |  |  |
|  | |  |  |  |  |
|  | | 17.Was the sample treated?: | Categorical |  |  |
|  | | a. Treated |  |  |  |
|  | | b. Not treated |  |  |  |
|  | | c. Mixed |  |  |  |
|  | | d. N/R |  |  |  |
|  | |  |  |  |  |
|  | | 18. What is the criminal justice status? | Categorical |  |  |
|  | | a. Arrested |  |  |  |
|  | | b. Charged |  |  |  |
|  | | c. Pre-trial/detained |  |  |  |
|  | | d. Adjudicated/sentenced |  |  |  |
|  | | e. Pre-disposition |  |  |  |
|  | | f. Mixed |  |  |  |
|  | | g. Unknown |  |  |  |
|  | |  |  |  |  |
|  | | 19. What is the mean age and standard deviation? | Numeric |  |  |
|  | | a. Cis-females |  |  |  |
|  | | b. Cis-males  c. Gender/sexual minority |  |  |  |
|  | | (provide details in comments) |  |  |  |
|  | | 20. What is the ethnic breakdown of cis-females? | Categorical |  |  |
|  | | a. White |  |  |  |
|  | | b. Black |  |  |  |
|  | | c. Aboriginal |  |  |  |
|  | | d. Hispanic |  |  |  |
|  | | d. Asian |  |  |  |
|  | | e. Multiracial |  |  |  |
|  | | f. Other |  |  |  |
|  | |  |  |  |  |
|  | | 21. What is the ethnic breakdown of cis-males? | Categorical |  |  |
|  | | a. White |  |  |  |
|  | | b. Black |  |  |  |
|  | | c. Aboriginal |  |  |  |
|  | | d. Hispanic |  |  |  |
|  | | d. Asian |  |  |  |
|  | | e. Multiracial |  |  |  |
|  | | f. Other |  |  |  |
|  | | 22. What is the ethnic breakdown of sexual/gender minorities?  (provide details in comments) |  |  |  |
|  | | 23. Index type crime for justice-involved cis-females: | Categorical |  |  |
|  | | a. Violent |  |  |  |
|  | | b. Sexual |  |  |  |
|  | | c. Non-violent |  |  |  |
|  | | d. Drug |  |  |  |
|  | | e. Other |  |  |  |
|  | |  |  |  |  |
|  | | 24. Index type crime for justice-involved cis-males: | Categorical |  |  |
|  | | a. Violent |  |  |  |
|  | | b. Sexual |  |  |  |
|  | | c. Non-violent |  |  |  |
|  | | d. Drug |  |  |  |
|  | | e. Other |  |  |  |
|  | | 25. Index type crime for justice-involved of sexual/gender minorities  (provide details in comments)  a. Violent  b. Sexual  c. Non-violent  d. Drug  e. Other | Categorical |  |  |
|  | |  |  |  |  |
|  | | 26. What is the risk level of the overall sample? | Categorical |  |  |
|  | | a. Low |  |  |  |
|  | | b. Moderate |  |  |  |
|  | | c. High |  |  |  |
|  | | d. Not applicable |  |  |  |
|  | | e. Not reported |  |  |  |
| Risk of bias | |  |  |  |  |
|  | | 27. Study population description. Does the document describe the source population in replicable detail? |  |  |  |
|  | | a. Yes |  |  |  |
|  | | b. No |  |  |  |
|  | | c. Unclear |  |  |  |
|  | |  |  |  |  |
|  | | 28. Study population criteria: Does the document list all inclusion and exclusion criteria for participation? | Categorical |  |  |
|  | | a. Yes |  |  |  |
|  | | b. No |  |  |  |
|  | | c. Unclear |  |  |  |
|  | |  |  |  |  |
|  | | 29. Prospective study: Was the study prospective (i.e., the sample was selected prior to the onset of offending? | Y/N |  |  |
|  | | a. Yes |  |  |  |
|  | | b. No |  |  |  |
|  | | c. Unclear |  |  |  |
|  | | 30. Outcome descriptor: Was the criteria for offending/recidivism described in replicable detail? | Y/N |  |  |
|  | | a. Yes |  |  |  |
|  | | b. No |  |  |  |
|  | | c. Unclear |  |  |  |
|  | | 31. Risk factor description: Were all factors described in replicable detail? | Y/N |  |  |
|  | | a. Yes |  |  |  |
|  | | b. No |  |  |  |
|  | | c. Unclear |  |  |  |
|  | | 32. Risk factor validity: Were all measures of the risk factor based on a validated measure? | Y/N |  |  |
|  | | a. Yes |  |  |  |
|  | | b. No |  |  |  |
|  | | c. Unclear |  |  |  |
|  | | 33. Risk factor timing: Were all factors either measured before the onset of offending/recidivism, or measured retrospectively to a time prior to offending? | Y/N |  |  |
|  | | a. Yes |  |  |  |
|  | | b. No |  |  |  |
|  | | c. Unclear |  |  |  |
|  | |  |  |  |  |
|  | | 34. Selective risk factor reporting: was the study free from reporting bias? | Y/N |  |  |
|  | | a. Yes |  |  |  |
|  | | b. No |  |  |  |
|  | | c. Unclear |  |  |  |
|  | |  |  |  |  |
|  | | 35. Selective analysis reporting: was the study free from analysis reporting bias? | Y/N |  |  |
|  | | a. Yes |  |  |  |
|  | | b. No |  |  |  |
|  | | c. Unclear |  |  |  |
|  | |  |  |  |  |
| Risk factors | | (Complete for each factor reported) |  |  |  |
|  | | 36. Risk factor | Text |  |  |
|  | | 37. Conceptual definition of risk factor | Text |  |  |
|  | | 38.Operational definition | Text |  |  |
|  | | 39. Origin of the factor’s variable: | Categorical |  |  |
|  | | a. Official data (government/police) |  |  |  |
|  | | b. Self-reported |  |  |  |
|  | | c. Peer-reported |  |  |  |
|  | | d. Family-reported |  |  |  |
|  | | e. Practitioner-reported (including school) |  |  |  |
|  | | f. Other |  |  |  |
|  | |  |  |  |  |
|  | | 40. Measured retrospectively? | Y/N |  |  |
|  | | a. Yes |  |  |  |
|  | | b. No |  |  |  |
|  | | c. Unclear |  |  |  |
|  | | 41. Age group associated with risk factor | Categorical |  |  |
|  | | a. Under 15 years |  |  |  |
|  | | b. 15-18 years |  |  |  |
|  | | c. 18-21 years |  |  |  |
|  | | d. Over 21 years |  |  |  |
|  | | e. Other age categorization |  |  |  |
|  | |  |  |  |  |
|  | | 42. Risk factor domain: | Categorical |  |  |
|  | | a. Socio-demographic |  |  |  |
|  | | b. Social |  |  |  |
|  | | c. Economic |  |  |  |
|  | | d. Psychological |  |  |  |
|  | | e. Experiential |  |  |  |
|  | | f. Environmental |  |  |  |
|  | | f. Other |  |  |  |
|  | |  |  |  |  |
|  | | 43. Did a test of statistical significance indicate statistically significant differences between the offending and non-offending groups(s)? | Categorical |  |  |
|  | | a. Yes |  |  |  |
|  | | b. No |  |  |  |
|  | | c. Can’t tell |  |  |  |
|  | | d. N/A (no testing completed) |  |  |  |
|  | |  |  |  |  |
|  | | 44. Was a standardized effect size reported? | Y/N |  |  |
|  | | a. Yes |  |  |  |
|  | | b. No |  |  |  |
|  | | If Yes: | Text |  |  |
|  | |  |  |  |  |
|  | | 45. Effect size measure | Categorical |  |  |
|  | | a. r |  |  |  |
|  | | b. b |  |  |  |
|  | | c. B(exp) |  |  |  |
|  | | d. OR |  |  |  |
|  | | e. Other |  |  |  |
|  | |  |  |  |  |
|  | | 46. Effect size | Numerical |  |  |
|  | | 47. Standard error of effect size | Numerical |  |  |
|  | | 48. Effect size reported on page number | Numerical |  |  |
|  | |  |  |  |  |
|  | | 49. If no to Q43, are data available to calculate effect size? | Y/N |  |  |
|  | | d. Yes |  |  |  |
|  | | e. No |  |  |  |
|  | |  |  |  |  |
|  | | 50. If yes to Q49, type of data effect size can be calculated from: | Categorical |  |  |
|  | | a. Means and standard deviations |  |  |  |
|  | | b. Frequencies or proportions (dichotomous) |  |  |  |
|  | | c. Frequencies or proportions (polychotomous) |  |  |  |
|  | | d. Unadjusted correlation coefficient |  |  |  |
|  | | e. Multiple regression coefficients (unstandardized) |  |  |  |
|  | | f. Multiple regression coefficients (standardized) |  |  |  |
|  | | g. Odds ratio(s) |  |  |  |
|  | | h. t-value or F-value |  |  |  |
|  | | i. Chi-square (df=1) |  |  |  |
|  | | j. Other (specify) |  |  |  |
|  | |  |  |  |  |
|  | | **Means and Standard Deviations** |  |  |  |
|  | | 51. Offending group mean | Numerical |  |  |
|  | | 52.Comparison group mean | Numerical |  |  |
|  | | 53.Offending group standard deviation | Numerical |  |  |
|  | | 54. Comparison group standard deviation | Numerical |  |  |
|  | |  |  |  |  |
|  | | **Proportions or frequencies** |  |  |  |
|  | | 55. n of offending with the risk factor(s) | Numerical |  |  |
|  | | 56.n of comparison group with the risk factor(s) | Numerical |  |  |
|  | | 57. Proportion of offending group with the risk factor | Numerical |  |  |
|  | | 58.Proportion of comparison group with the risk factor | Numerical |  |  |
|  | |  |  |  |  |
|  | | **Regression coefficients and correlations** |  |  |  |
|  | | 59.Unadjusted correlation coefficient | Numerical |  |  |
|  | | 60.Standardized regression coefficient | Numerical |  |  |
|  | | 61.Unstandardized regression coefficient | Numerical |  |  |
|  | | 62.Standard deviation of predictor | Numerical |  |  |
|  | | 63.Control variables | Numerical |  |  |
|  | |  |  |  |  |
|  | | **Significance Tests** |  |  |  |
|  | | 64.t-value | Numerical |  |  |
|  | | 65.F-value | Numerical |  |  |
|  | | 66.Chi-square value (df=1) | Numerical |  |  |
|  | | **Calculated Effect Size** |  |  |  |
|  | | 67.Effect size | Numerical |  |  |
|  | | 68.Standard error of effect size | Numerical |  |  |
| Strength factors | | (Complete for each factor reported) |  |  |  |
|  | | 69. Strength factor | Text |  |  |
|  | | 70. Conceptual definition of strength factor | Text |  |  |
|  | | 71.Operational definition | Text |  |  |
|  | | 72. Origin of the factor’s variable: | Text |  |  |
|  | | a. Official data (government/police) | Categorical |  |  |
|  | | b. Self-reported |  |  |  |
|  | | c. Peer-reported |  |  |  |
|  | | d. Family-reported |  |  |  |
|  | | e. Practitioner-reported (including school) |  |  |  |
|  | | f. Other |  |  |  |
|  | |  |  |  |  |
|  | | 73. Measured retrospectively? | Y/N |  |  |
|  | | a. Yes |  |  |  |
|  | | b. No |  |  |  |
|  | | c. Unclear |  |  |  |
|  | | 74. Age group associated with strength factor | Categorical |  |  |
|  | | a. Under 15 years |  |  |  |
|  | | b. 15-18 years |  |  |  |
|  | | c. 18-21 years |  |  |  |
|  | | d. Over 21 years |  |  |  |
|  | | e. Other age categorization |  |  |  |
|  | |  |  |  |  |
|  | | 75. Strength factor domain: | Categorical |  |  |
|  | | a. Socio-demographic |  |  |  |
|  | | b. Social |  |  |  |
|  | | c. Economic |  |  |  |
|  | | d. Psychological |  |  |  |
|  | | e. Experiential |  |  |  |
|  | | f. Environmental |  |  |  |
|  | | g. Other |  |  |  |
|  | |  |  |  |  |
|  | | 76. Did a test of statistical significance indicate statistically significant differences between the offending and non-offending groups(s)? | Categorical |  |  |
|  | | a. Yes |  |  |  |
|  | | b. No |  |  |  |
|  | | c. Can’t tell |  |  |  |
|  | | d. N/A (no testing completed) |  |  |  |
|  | |  |  |  |  |
|  | | 77. Was a standardized effect size reported? | Y/N |  |  |
|  | | a. Yes |  |  |  |
|  | | b. No |  |  |  |
|  | | If Yes: | Numerical |  |  |
|  | |  |  |  |  |
|  | | 78. Effect size measure | Categorical |  |  |
|  | | a. r |  |  |  |
|  | | b. b |  |  |  |
|  | | c. B(exp) |  |  |  |
|  | | d. OR |  |  |  |
|  | | e. Other |  |  |  |
|  | |  |  |  |  |
|  | | 79. Effect size | Numerical |  |  |
|  | | 80. Standard error of effect size | Numerical |  |  |
|  | | 81. Effect size reported on page number | Numerical |  |  |
|  | |  |  |  |  |
|  | | 82. If no to Q76, are data available to calculate effect size? | Y/N |  |  |
|  | | d. Yes |  |  |  |
|  | | e. No |  |  |  |
|  | |  |  |  |  |
|  | | 83. If yes to Q76, type of data effect size can be calculated from: | Categorical |  |  |
|  | | a. Means and standard deviations |  |  |  |
|  | | b. Frequencies or proportions (dichotomous) |  |  |  |
|  | | c. Frequencies or proportions (polychotomous) |  |  |  |
|  | | d. Unadjusted correlation coefficient |  |  |  |
|  | | e. Multiple regression coefficients (unstandardized) |  |  |  |
|  | | f. Multiple regression coefficients (standardized) |  |  |  |
|  | | g. Odds ratio(s) |  |  |  |
|  | | h. t-value or F-value |  |  |  |
|  | | i. Chi-square (df=1) |  |  |  |
|  | | j. Other (specify) |  |  |  |
|  | |  |  |  |  |
|  | | **Means and Standard Deviations** |  |  |  |
|  | | 84. Offending group mean | Numerical |  |  |
|  | | 85.Comparison group mean | Numerical |  |  |
|  | | 86.Offending group standard deviation | Numerical |  |  |
|  | | 87. Comparison group standard deviation | Numerical |  |  |
|  | |  |  |  |  |
|  | | **Proportions or frequencies** |  |  |  |
|  | | 88. n of offending with the risk factor(s) | Numerical |  |  |
|  | | 89. n of comparison group with the risk factor(s) | Numerical |  |  |
|  | | 90. Proportion of offending group with the risk factor | Numerical |  |  |
|  | | 91.Proportion of comparison group with the risk factor | Numerical |  |  |
|  | |  |  |  |  |
|  | | **Regression coefficients and correlations** |  |  |  |
|  | | 92.Unadjusted correlation coefficient | Numerical |  |  |
|  | | 93.Standardized regression coefficient | Numerical |  |  |
|  | | 94.Unstandardized regression coefficient | Numerical |  |  |
|  | | 95. Standard deviation of predictor | Numerical |  |  |
|  | | 96.Control variables | Numerical |  |  |
|  | |  |  |  |  |
|  | | **Significance Tests** |  |  |  |
|  | | 97.t-value | Numerical |  |  |
|  | | 98.F-value | Numerical |  |  |
|  | | 99.Chi-square value (df=1) | Numerical |  |  |
|  | | **Calculated Effect Size** |  |  |  |
|  | | 100.Effect size | Numerical |  |  |
|  | | 101.Standard error of effect size | Numerical |  |  |
|  | |  |  |  |  |
| Authors’ conclusion | |  |  |  |  |
|  | | 103. What did the authors conclude about the relationship? | Categorical/Text |  |  |
|  | | a. Risk/strength factor increases likelihood of offending |  |  |  |
|  | | b. Risk/strength factor reduces the likelihood of offending |  |  |  |
|  | | c. No effect of risk/strength factor on offending |  |  |  |
|  | | d. Unclear/no conclusion stated by authors |  |  |  |
|  | |  |  |  |  |
|  | |  |  |  |  |
|  | |  |  |  |  |
|  | |  |  |  |  |
|  | |  |  |  |  |
